# Genome Sequence of *Bradyrhizobium japonicum* E109, One of the Most Agronomically Used Nitrogen-Fixing Rhizobacteria in Argentina

**DOI:** 10.1128/genomeA.01566-14

**Published:** 2015-02-19

**Authors:** Daniela Torres, Santiago Revale, Melissa Obando, Guillermo Maroniche, Gastón Paris, Alejandro Perticari, Martín Vazquez, Florence Wisniewski-Dyé, Francisco Martínez-Abarca, Fabricio Cassán

**Affiliations:** a Universidad Nacional de Rio Cuarto (UNRC), Córdoba, Argentina; b Instituto de Agrobiotecnología Rosario (INDEAR), Rosario, Argentina; c Instituto de Microbiología y Zoología Agrícola-Instituto Nacional de Tecnología Agropecuaria (IMYZA-INTA), Castelar, Buenos Aires, Argentina; d Instituto Leloir, Buenos Aires, Argentina; e Ecologie Microbienne, Université Lyon 1, Villeurbanne, France; f Grupo de Ecología Genética de la Rizósfera, Estación Experimental del Zaidín (CSIC), Granada, Spain

## Abstract

We present here the complete genome sequence of *Bradyrhizobium japonicum* strain E109, one of the most used rhizobacteria for soybean inoculation in Argentina since the 1970s. The genome consists of a 9.22-Mbp single chromosome and contains several genes related to nitrogen fixation, phytohormone biosynthesis, and a rhizospheric lifestyle.

## GENOME ANNOUNCEMENT

The soybean-Bradyrhizobium symbiosis is considered one of the most efficient in fixing N_2_ and probably the greatest in economic importance around the world (1). The agronomic cultivation of soybeans (*Glycine max* L.) affects >20 million ha in Argentina, where at least 85%, on average, are biologically treated with bradyrhizobia (2). In the 1970s, several strains belonging to the genus Bradyrhizobium were received in Argentina from different collections around the world to be evaluated under agronomical conditions, including strain 2860 (previously named USDA138 and corresponding to the *Bradyrhizobium japonicum* USDA6 serogroup), sent by C. N. Hale from the Department of Scientific and Industrial Research (DSIR) in New Zealand. After evaluation, selection, and reisolation from soybean nodules, the strain was renamed E109. To date, *B. japonicum* E109 has been the only strain recommended by the Instituto Nacional de Tecnología Agropecuaria (INTA) for soybean inoculation due to its capacity to effectively colonize the plant and fix nitrogen, increasing crop productivity (3). Together with this ability, alternative mechanisms have been proposed to explain the growth promotion, especially in nonlegumes, such as phosphate solubilization (4), siderophore production (5), systemic resistance induction (6), and phytohormone biosynthesis (7–9).

We announce here the complete annotated genome sequence of *B. japonicum* E109. The sequence was obtained using a combined whole-genome shotgun and 8-kb paired-end strategy with a 454 GS FLX Titanium pyrosequencer at the Instituto de Agrobiotecnología Rosario (INDEAR) (Argentina), resulting in a 24-fold genome coverage. The sequencing reads were *de novo* assembled (Newbler version 2.9), resulting in 142 contigs ordered in 4 scaffolds (>737 kbp each; *N*_50_, 4,081,299 bp). Intra- and interscaffold gap closures were achieved by a detailed observation of the relevant sequencing reads using the Geneious R7 software platform (10). The absence of plasmids is a common feature of Bradyrhizobium genomes (11–13), and in agreement with the bioinformatics data, pulsed-field gel electrophoresis (PFGE) analysis of total DNA revealed the presence of a unique chromosome. The genome size is 9,224,208 bp, and the G+C content, in agreement with this bacterial species, is 63.6%.

Genome annotation was done using the NCBI Prokaryotic Genomes Annotation Pipeline (PGAP) (14). The complete genome consists of 8.233 protein-coding sequences. Similarly to other species of the Bradyrhizobium genus, *B. japonicum* E109 contains two identical and complete ribosomal operons. A total of 54 tRNA genes representing 45 tRNA species were identified. In agreement with the genome sequence of USDA6, the presence of a 645-kb DNA region with low G+C content (59.0%) was revealed to be reminiscent of a symbiotic island, which includes most of the *nod*, *nif*, and *fixes* genes. The putative genes involved in other plant growth-promoting mechanisms, such as phytohormone production, were determined using the RAST annotation server (15) and KAAS (16).

The *B. japonicum* E109 genome contains genes related to type II and VI secretion systems, nitrogen fixation, phytohormone biosynthesis, and a rhizospheric lifestyle. The genome sequence of E109 provides a genomic basis for in-depth comparative genome analyses to elucidate the specific mechanisms of Bradyrhizobium-plant interactions.

### Nucleotide sequence accession number.

The complete genome sequence of *B. japonicum* E109 is available at NCBI GenBank under the accession no. CP010313.
